# Independent Origins of New Sex-Linked Chromosomes in the *melanica *and *robusta *Species Groups of *Drosophila*

**DOI:** 10.1186/1471-2148-8-33

**Published:** 2008-01-29

**Authors:** Sergio V Flores, Amy L Evans, Bryant F McAllister

**Affiliations:** 1 Department of Biological Sciences & Roy J. Carver Center for Comparative Genomics, 143 Biology Building, University of Iowa, Iowa City, IA 52242, USA

## Abstract

**Background:**

Recent translocations of autosomal regions to the sex chromosomes represent important systems for identifying the evolutionary forces affecting convergent patterns of sex-chromosome heteromorphism. Additions to the sex chromosomes have been reported in the *melanica *and *robusta *species groups, two sister clades of *Drosophila*. The close relationship between these two species groups and the similarity of their rearranged karyotypes motivates this test of alternative hypotheses; the rearranged sex chromosomes in both groups are derived through a common origin, or the rearrangements are derived through at least two independent origins. Here we examine chromosomal arrangement in representatives of the *melanica *and the *robusta *species groups and test these alternative hypotheses using a phylogenetic approach.

**Results:**

Two mitochondrial and two nuclear gene sequences were used to reconstruct phylogenetic relationships of a set of nine ingroup species having fused and unfused sex chromosomes and representing a broad sample of both species groups. Different methods of phylogenetic inference, coupled with concurrent cytogenetic analysis, indicate that the hypothesis of independent origins of rearranged sex chromosomes within each species group is significantly more likely than the alternative hypothesis of a single common origin. An estimate tightly constrained around 8 My was obtained for the age of the rearranged sex chromosomes in the *melanica *group; however, a more loosely constrained estimate of 10–15 My was obtained for the age of the rearrangement in the *robusta *group.

**Conclusion:**

Independent acquisition of new chromosomal arms by the sex chromosomes in the *melanica *and *robusta *species groups represents a case of striking convergence at the karyotypic level. Our findings indicate that the parallel divergence experienced by newly sex-linked genomic regions in these groups represents an excellent system for studying the tempo of sex chromosome evolution.

## Background

Pairs of sex chromosomes generally display high structural and functional differentiation. Heteromorphism between the sex-chromosome pair in different lineages was originally proposed as resulting from convergence, whereby presence of the primary locus controlling sex determination on a pair of homologous chromosomes defines a characteristic pattern of differentiation [1-3]. Autosomal origins of independently derived sex chromosomes have now been clearly demonstrated in humans [4,5], birds [6], snakes [7] and in several lineages of plants [8]. Convergence upon similar patterns of heteromorphism in a variety of organisms indicates that common mechanisms shape the evolution of sex chromosomes [9-12]; however, the rate at which heteromorphism develops on a newly derived sex-chromosome pair is unknown, as are the mechanisms affecting this transition.

Organisms with sex chromosomes in transitory stages of differentiation provide models for testing hypotheses relating to sex-chromosome evolution. Since the transition from an autosomal pair to fully differentiated heteromorphic sex chromosomes is a dynamic process that lasts several million years, studies of sex chromosomes from a broad spectrum of stages are ultimately needed to develop a synthetic picture of this process. Sex chromosomes underlying recent transitions to dioecy in different plant lineages represent promising sources of model systems [8,13]. Rearrangements involving translocations onto existing sex chromosomes represent another source for studying this evolutionary transition. Sex-linked transmission results from this type of translocation, and the newly acquired region of the sex chromosomes differentiates through the same evolutionary pathway as did the original pair of sex chromosomes; i.e., suppression of recombination, degeneration of the heterozygous chromosomes and dosage compensation [14,15]. In fact, the sex chromosomes in humans are a mosaic comprised of an ancestral sex-linked region shared with non-eutherian mammals and a newly acquired region unique to eutherian mammals [16].

A rich history of comparative genomics in the genus *Drosophila *makes this group especially appealing for studies of sex chromosome evolution. This appeal derives from several features: i) phylogenetic relationships among several major groups, and even within some groups, are generally understood, ii) extensive chromosomal analyses have been performed within many species groups, and iii) long-distance comparisons demonstrate a high degree of conservation in gene content within chromosomal arms. Therefore, the chromosomal arm designated as element A comprises the X chromosome in most extant species and contains a conserved set of genes [17,18]. The four major autosomal arms are designated as elements B, C, D, and E, and the "dot" as element F [19,20]. Comparative studies reveal several instances of translocations of these autosomal elements to the sex chromosomes, thus establishing newly sex-linked genomic regions subject to same forces that transform primary sex chromosomes.

Three independent examples of derived sex-linked regions in the genus *Drosophila *representing a wide spectrum in the transition from autosome to heteromorphic sex chromosomes have been the primary targets of research efforts to understand the mechanisms underlying sex chromosome evolution. At one extreme, an ancient centromeric fusion between the X chromosome and an autosome, corresponding to element D, occurred in the lineage leading to *D. pseudoobscura *and relatives [21]. The newly acquired arm of the metacentric X chromosome has co-opted the standard mechanism of dosage compensation utilized by the ancestral X [22,23]. Interestingly, this fusion is associated with translocation of Y-chromosome genes onto an autosome, so these previously male-limited genes now segregate as diploid autosomal loci [24]. Origin and gene content of the single Y chromosome in the *D. pseudoobscura *lineage remains unclear, but it potentially represents the degenerate homolog of the derived arm of the X. Transitional sex chromosomes at an intermediate stage of differentiation are present in *D. miranda*. This species has an enlarged Y chromosome due to a fusion between the Y and element C. The homolog of the neo-Y region segregates as a secondary X (or neo-X) chromosome. Gene function has been lost for some loci within the newly acquired region of the Y [25-28] and dosage compensation appears to have arisen regionally on the neo-X chromosome [22,23,29]. An even younger sex-linked region is present in *D. americana*, where element B is fused at the centromere with the X forming a neo-X chromosome. This rearrangement remains polymorphic with the ancestral unfused chromosomes, so Y-linked transmission of element B is transient and meiotic exchange still occurs between the neo-X and these transient neo-Y chromosomes [30,31]. Genes on the neo-Y chromosome are functional [32], dosage compensation is not evident [22,23], and sequence divergence is accruing specifically in association with a recombination-suppressing inversion complex limited to the neo-X chromosome [33,34]. Overall, these three rearrangements represent distinct time points in the transition from autosome to heteromorphic sex chromosomes, but they only provide a coarse measure of this progression.

Other cases of fusions involving sex chromosomes and autosomes have been reported in the genus *Drosophila*. For example, a fusion of the X chromosome with an autosome has been described in *D. robusta *and relatives in the *robusta *species group [35,36]. This derived arm of the X originated through a centromeric fusion with element D [37-39]. The X chromosome of *D. melanica *also has two arms (XL and XR), indicating a fusion between an autosome and the ancestral X [40]. Other members of the *melanica *species group (*D. paramelanica *[41], *D. euronotus *[42], *D. nigromelanica *[43] and *D. melanura *[44]) exhibit the same rearrangement of the X, but the ancestral arrangement is retained in *D. micromelanica *[44]. Comparisons of polytene chromosomes also indicate homology to element D for the newly derived XL arm within the melanica group [45]. These two species groups are both contained within the *virilis*-*repleta *radiation of the subgenus *Drosophila*, and they are generally recognized as sister groups based on their high affinity at morphological [46], chromosomal [40,45] and molecular [47-49] levels. The close relationship between these species groups, and the similarity between the chromosomal rearrangements in each group, raises the possibility that the derived arrangements of the sex chromosomes share a common origin.

**Figure 1:**
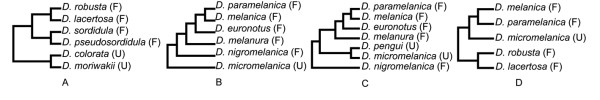
**Phylogenies proposed in the melanica and robusta groups**. Phylogenies previously reported for species in the *robusta *and *melanica *species groups. a) Relationships within the *robusta *group proposed by Narayanan [33], based on polytene chromosomes; b) and c) Alternative topologies proposed by Stalker ([41, 42], respectively) for species in the *melanica *group. F and U labels indicate karyotypes with fused or unfused sex chromosomes.

Phylogenetic relationships within and between the *robusta *and *melanica *species groups are not resolved. Narayanan [36] proposed relationships within the *robusta *species group based on analyses of polytene chromosomes. Two species with unfused sex chromosomes, *D. colorata *and *D. moriwakii*, were placed as basal members of the group relative to *D. robusta, D. lacertosa, D. sordidula *and *D. pseudosordidula*, all of which contain the derived fusion of the sex chromosomes (Fig. 1A). However, a subsequent taxonomic revision reassigned *D. colorata *and *D. moriwakii *to the *melanica *group [50]. Polytene chromosomes were also used by Stalker [44] to infer the relationships within the *melanica *species group. The ancestral unfused sex-chromosome arrangement was inferred for the basal lineage represented by *D. micromelanica *and the derived arrangement was inferred for *D. paramelanica, D. melanica, D. euronotus, D. melanura *and *D. nigromelanica *(Fig. 1B). However, in a subsequent analysis of chromosomal relationships, Stalker [45] suggested an alternative phylogeny for the *melanica *group where the divergence of *D. nigromelanica *represented the most basal node (Fig. 1C). He proposed that the alternative arrangements of the sex chromosomes, currently observed among the species of the *melanica *group, arose from a polymorphic population that existed for an extended period of time. Such a situation currently exists in *D. americana*, where the frequency of a segregating X-autosome fusion is correlated with latitude [30]. Phylogenetic relationships of species with the ancestral arrangement of the sex chromosomes (*D. colorata*, *D. moriwakii *and *D. micromelanica*) relative to species containing the rearranged sex chromosomes are critical for determining the number of rearrangements involved.

Considering the close relationship between the *robusta *and *melanica *species groups, and the high degree of similarity of their rearranged sex chromosomes, concurrent cytogenetic and phylogenetic analyses are needed to establish the evolutionary history of these rearrangements within these two species groups. Here we present results of a phylogenetic analysis of DNA sequences from two mitochondrial and two nuclear loci on the ancestral X chromosome from select species belonging to the *robusta *and *melanica *species groups and from appropriate outgroups together with an examination of sex-chromosome morphology. The species were selected based on previous reports of chromosomal morphology and availability of live material. Under the assumption that centromeric fusion is more likely than fission, these data provide a robust test of the alternative hypotheses of a single origin versus independent origins of the rearranged sex chromosomes in these sister groups of *Drosophila*. Based on the findings, at least two independent fusions with the sex chromosomes are clearly resolved. Furthermore, the results provide a phylogenetic framework for designing future studies of these newly sex-linked regions to test hypotheses relevant to mechanisms of sex chromosome evolution.

## Results

### Chromosomal arrangement in female and male karyotypes

**Figure 2:**
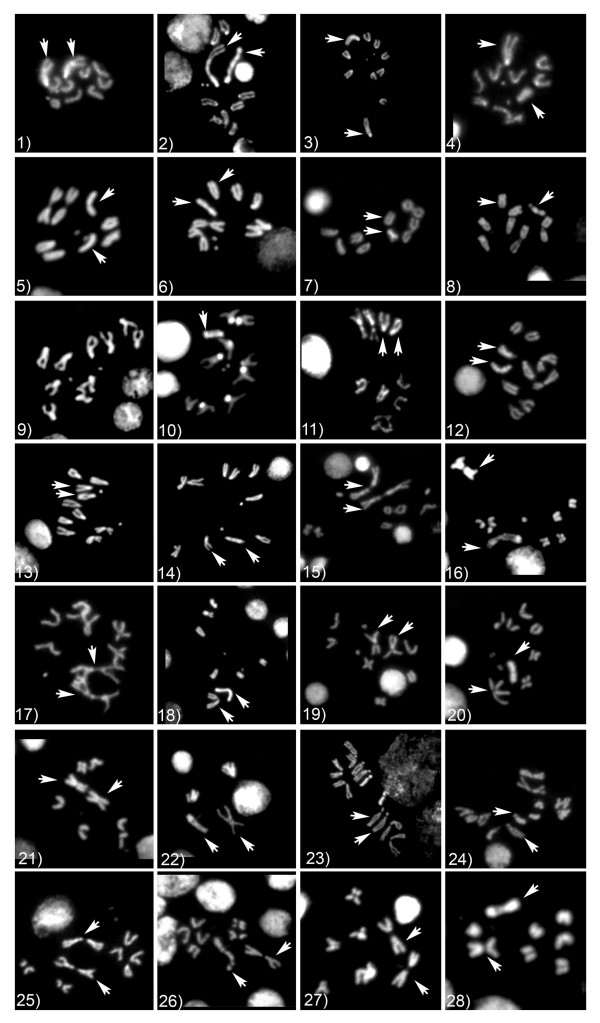
**Metaphase chromosomes in the species used in this study**. 1) – 2) *D. funebris *(female and male); 3) – 4) *D. macrospina*; 5) – 6) *D. pavani*; 7) – 8) *D. gaucha*; 9) – 10) *D. virilis*; 11) – 12) *D. borealis*; 13) – 14) *D. micromelanica*; 15) – 16) *D. nigromelanica*; 17) – 18) *D. euronotus*; 19) – 20) *D. paramelanica*; 21) – 22) *D. melanica*; 23) – 24) *D. colorata*; 25) – 26) *D. robusta*; 27) – 28) *D. sordidula*. Arrowheads indicate the identifiable sex chromosomes.

Coordinated analyses of chromosomal arrangements and phylogenetic relationships were performed in this study. Figure 2 shows metaphase plates for each of the species. Descriptions of the karyotypes in each of the species analyzed in this study are provided in Table 1. A fusion between an autosomal element and the sex chromosomes in *D. robusta *and *D. sordidula*, representatives of the *robusta *species group, and in *D. melanica*, *D. nigromelanica*, *D. euronotus *and *D. paramelanica*, representatives of the *melanica *species group, was confirmed by this analysis. The fusion is indicated by the presence of long metacentric X and Y chromosomes in contrast with the karyotype of outgroup species, which have acrocentric X and Y chromosomes similar to the inferred ancestral karyotype for the genus *Drosophila *[51]. Variation in the number of chromosomes (2n = 8, 10 and 12) while maintaining 14 chromosomal arms (represented by the Fundamental Number, or FN in Table 1)-except in *D. colorata *which has 16 chromosomal arms- also provided evidence supporting the X-autosome/Y-autosome fusion in the *melanica *and *robusta *groups. The increase in chromosomal arms in species of the *melanica *and *robusta *groups, in relation to the ancestral karyotype of *Drosophila *(FN = 12), is due to a pericentric inversion of the Muller's element B, previously reported in species belonging to both groups, with the exception of *D. pseudosordidula *[36,44], which was not analyzed in this study. The inverted chromosome consists of the small metacentric chromosomal pair in each of the karyotypes from species in the *melanica *and *robusta *species groups (See Fig. 2). Here we confirm that this is a synapomorphic rearrangement shared by the members of the *melanica*-*robusta *clade.

**Table 1:** Features of Metaphase Karyotypes from Ingroup and Outgroup Species

Group	Species	2n	M	A	D	FN	X	Y
*robusta*	*D. sordidula*	10	2	2	1	14	Longer than autosomes, metacentric	Longer than the X, submetacentric,
	*D. robusta*	8	3	-	1	14	Longer than autosomes, metacentric	Longer than the X, submetacentric,
*melanica*	*D. colorata*	12	2	4	0	16	Similar size that autosomes, acrocentric.	Similar size that autosomes, acrocentric
	*D. micromelanica*	12	1	4	1	14	Similar size that autosomes, acrocentric.	Similar size that autosomes, acrocentric.
	*D. melanica*, *D. nigromelanica*, *D. euronotus *and *D. paramelanica*	10	2	2	1	14	Longer than autosomes, metacentric	Longer than autosomes,, submetacentric
*funebris*	*D. funebris*, *D. macrospina*	12	-	5	1	12	Longer than autosomes, acrocentric	Longer than autosomes, acrocentric
*mesophragmatica*	*D. pavani*, *D. gaucha*	10	1	3	1	12	Similar size that autosomes, acrocentric	Similar size that autosomes, acrocentric
*virilis*	*D. virilis*	12	-	5	1	12	Similar size that autosomes, acrocentric	Similar size that autosomes, acrocentric
	*D. borealis*	10	1	4	0	12		

Note: Each karyotype described by diploid number (2n), pairs of metacentric (M), acrocentric (A) and dot (D) chromosomes, and fundamental number (FN). Features of the sex chromosomes (X and Y) are summarized in terms of size and morphology.

Polytene chromosomes also reveal an independently derived pericentric inversion in Muller's element C of *D. colorata *[52], thus giving rise to the extra pair of chromosomal arms (FN = 16) in its karyotype (Fig. 2). In our analysis, the dot chromosome (element F) is absent in this species and is instead replaced by a large acrocentric chromosomal pair with intense DAPI staining. This is not consistent with the description of the metaphase karyotype reported by Narayan [36]. Our findings generally agree with the karyotype description of Wharton [53] and indicate that element F is enlarged in *D. colorata*, a phenomenon that has also occurred in *D. yooni *in the *diamphidiopoda *species group [54] and in *D. ananassae *in the *melanogaster *species group [55]. Excluding *D. colorata*, our descriptions of karyotype structure agree with the karyotype configurations reported for each species.

### Phylogenetic analysis of single partitions and combined data

**Figure 3:**
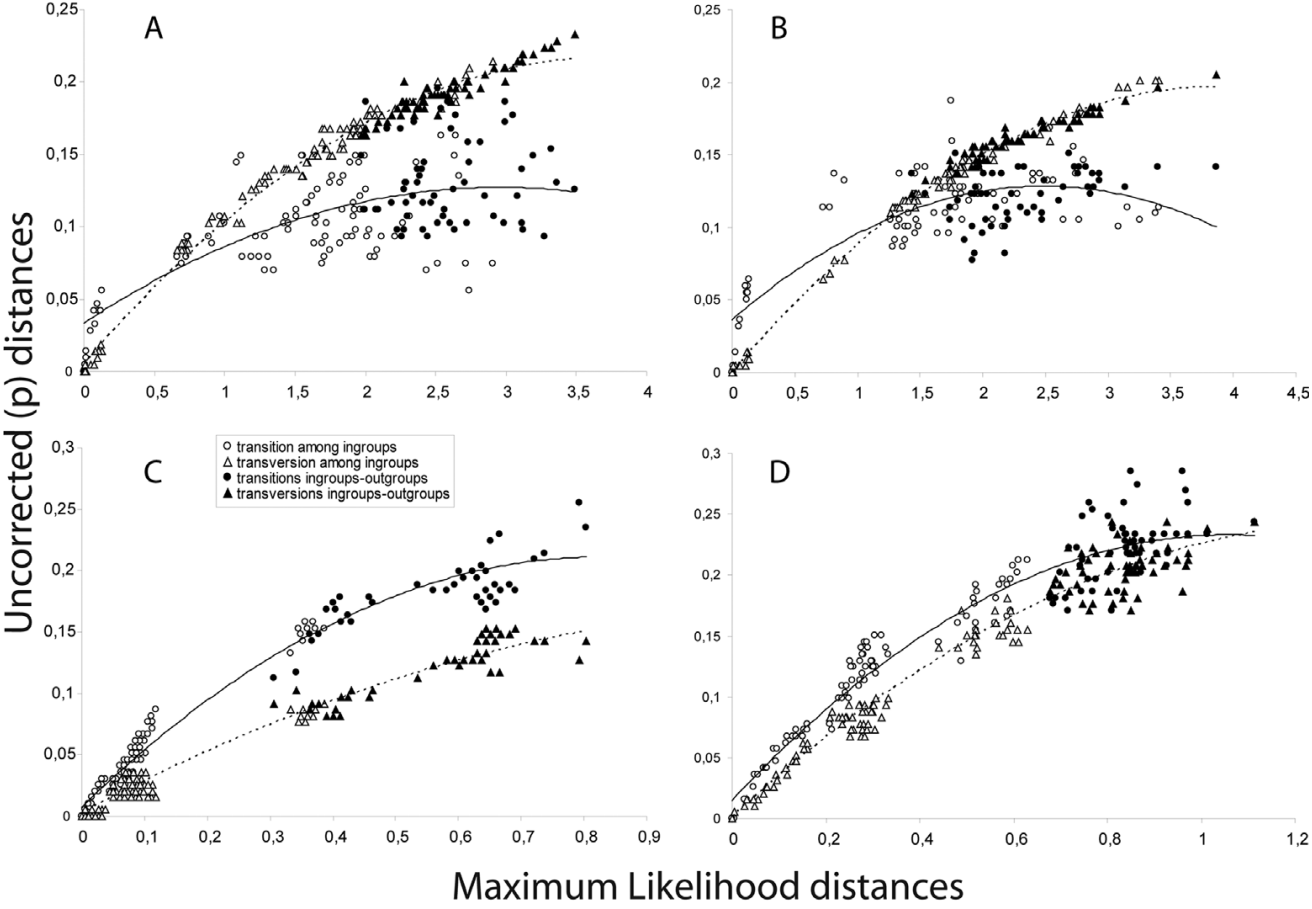
**Saturation on third codon positions**. Comparison between ML distance (X axis) and uncorrected distances for third codon positions (Y axis) estimated from: a) *CoI*, b) *CoII*, c) *cac*, and d) *sc *sequences. Transitions and transversions distances within ingroup and between ingroup-outgroup are labeled. Dotted lines correspond to regression lines fitted to transitions and solid lines for transversions.

Sequences from the mitochondrial genes *Cytochrome oxidase I *(*mtCoI*) and *Cytochrome oxidase II *(*mtCoII*), and from the X-chromosome gene regions *cacophony *(*cac*) and *scute *(*sc*) were used in the phylogenetic analysis. After excluding regions of *scute *with many indels among repetitious codons and introns from *cacophony*, the alignment matrix contained the following sizes: *CoI *= 645 bp; *CoII *= 657 bp; *cac *= 588 bp and *sc *= 579 bp. The mitochondrial genes (*CoI *and *CoII*) showed higher observed frequencies of transversions than transitions, even for short corrected pairwise-distances. This is a common pattern for mitochondrial genes in *Drosophila *[56], and is apparently due to rapid saturation for transition substitutions. Also, the corrected distances estimated from the mitochondrial sequences failed to separate outgroup from ingroup taxa (Fig. 3). Consequently, third positions of the *CoI *and *CoII *codons were removed in the following analyses. The nucleotide composition, substitution rates, transition-transversions ratios and models of nucleotide substitution for alternative partitions of the data are summarized in Table 2. Homogeneity tests showed similar phylogenetic signals for each major data partition, although the two nuclear genes did exhibit marginally non-significant heterogeneity (ILD test, *P *= 0.056). However, this weak incongruence between nuclear genes was not indicated when outgroups were removed (Table 3); therefore, the full concatenated dataset was used for hypothesis testing.

**Table 2:** Properties of Sequence Data Partitions Based on Sequenced Region, Codon Position and Genome Location

Data set	PIS	Empirical base frequency		Rates		**Ts:Tv rate (MP Tv:Ts)**^a^	Shape	Pinvar	ML Model
COI 1,2,3	165	A = 0.278 C = 0.146	G = 0.174 T = 0.402	A-C = 0.000 A-G = 21.995 A-T = 21.885	C-G = 0.000 C-T = 72.908 G-T = 1.000	2.287 (2:1)	2.100	0.631	GTR+I+G
COI 1,2	32	A = 0.201 C = 0.203	G = 0.243 T = 0.353	A-C = 0.000 A-G = 3.156 A-T = 4.787	C-G = 0.000 C-T = 72.078 G-T = 1.000	10.024 (10:1)	-	0.867	GTR+I
COI 3	133	A = 0.446 C = 0.071	G = 0.031 T = 0.448	A-C = 0.0000 A-G = 12.996 A-T = 0.207	C-G = 0.000 C-T = 4.894 G-T = 1.000	5.275 (5:1)	0.769	0.063	GTR+I+G
COII 1,2,3	158	A = 0.319 C = 0.118	G = 0.139 T = 0.424	A-C = 7.139 A-G = 24.723 A-T = 16.552	C-G = 0.000 C-T = 155.021 G-T = 1.000	4.489 (4:1)	2.676	0.647	GTR+I+G
CoII 1,2	32	A = 0.285 C = 0.158	G = 0.188 T = 0.370	A-C = 8.528 A-G = 10.570 A-T = 0.000	C-G = 0.000 C-T = 48.380 G-T = 1.000	7.542 (8:1)	-	0.873	GTR+I
COII 3	126	A = 0.431 C = 0.083	G = 0.025 T = 0.463	A-C = 0.4255 A-G = 38.165 A-T = 0.175	C-G = 0.000 C-T = 22.982 G-T = 1.000	6.592 (6:1)	0.516	0.223	HKY+G
Mt 1,2,3	323	A = 0.295 C = 0.134	G = 0.155 T = 0.417	A-C = 1.510 A-G = 22.441 A-T = 18.857	C-G = 0.000 C-T = 94.312 G-T = 1.000	3.045 (3:1)	2.113	0.638	GTR+I+G
Mt 1,2	64	A = 0.239 C = 0.185	G = 0.218 T = 0.359	A-C = 5.798 A-G = 13.824 A-T = 2.814	C-G = 0.000 C-T = 70.327 G-T = 1.000	8.718 (9:1)	-	0.876	GTR+I
cac	113	A = 0.224 C = 0.211	G = 0.224 T = 0.341	A-C = 4.291 A-G = 9.301 A-T = 1.607	C-G = 2.422 C-T = 9.301 G-T = 1.000	2.204 (2:1)	0.629	0.455	TVM+I+G
cac 3	97	A = 0.2481 C = 0.251	G = 0.277 T = 0.243	A-C = 2.416 A-G = 4.943 A-T = 1.835	C-G = 1.620 C-T = 4.874 G-T = 1.000	2.178 (2:1)	0.912	0.293	K80+G
sc	235	A = 0.236 C = 0.337	G = 0.252 T = 0.176	A-C = 1.000 A-G = 3.329 A-G = 2.094	C-G = 2.094 C-T = 4.472 G-T = 1.000	1.190 (1:1)	0.451	0.000	TIM+I+G
sc 3	127	A = 0.134 C = 0.134	G = 0.134 T = 0.410	A-C = 2.142 A-G = 3.837 A-T = 2.040	C-G = 0.000 C-T = 72.078 G-T = 1.000	1.516 (2:1)	2.247	0.124	HKY+G
Nu	348	A = 0.231 C = 0.276	G = 0.236 T = 0.257	A-C = 1.980 A-G = 5.834 A-G = 1.745	C-G = 1.670 C-T = 4.294 G-T = 1.000	1.447 (1:1)	0.285	0.000	TVM+G
Nu + mt 1,2,3	671	A = 0.266 C = 0.215	G = 0.190 T = 0.329	A-C = 2.873 A-G = 11.868 A-G = 8.568	C-G = 7.867 C-T = 20.930 G-T = 1.000	1.574 2:1	1.352	0.546	GTR+I+G
Nu + mt 1,2	412	A = 0.235 C = 0.244	G = 0.226 T = 0.295	A-C = 3.112 A-G = 8.2980 A-G = 1.912	C-G = 5.240 C-T = 10.717 G-T = 1.000	1.814 (2:1)	0.898	0.582	GTR+I+G

Note : Nucleotide composition, transformation rates and models of nucleotide change obtained from the Akaike Information Criterion test as implemented in ModelTest.^a ^Transversions (Tv)/Transitions (Ts) ratios used in differential weighting in the MP analysis. CoI = cytochrome oxidase I; CoII = cytochrome oxidase II, cac = cacophony; sc = scute; Mt = mitochondrial genes; Nu = nuclear genes; PIS = number of parsimony-informative sites; Pinvar = proportion of invariant sites; Shape = shape parameter of the gamma distribution. 1, 2 and 3 indicate first, second and third codon positions, respectively.

**Table 3:** Homogeneity Test of Data Partitions Based on Sequenced Region and Genome

Partitions	P (PIS) Ingroup only	P (PIS) Including outgroups
Between Mt genes (*CoI *vs *CoII*)	0.789 (43)	0.960 (64)
Between nuclear genes (*cac vs sc*)	0.918 (110)	0.056 (347) *
Mt vs nuclear genes	0.799 (153)	0.879 (411)
Among all genes (*CoI, CoII, cac, sc*)	0.885 (153)	0.124 (411)

Note: The asterisk indicates marginal significant difference among the phylogenetic signals from the respective partition. P and PIS indicates probability derived from the homogeneity test and number of parsimony-informative sites, respectively.

**Figure 4:**
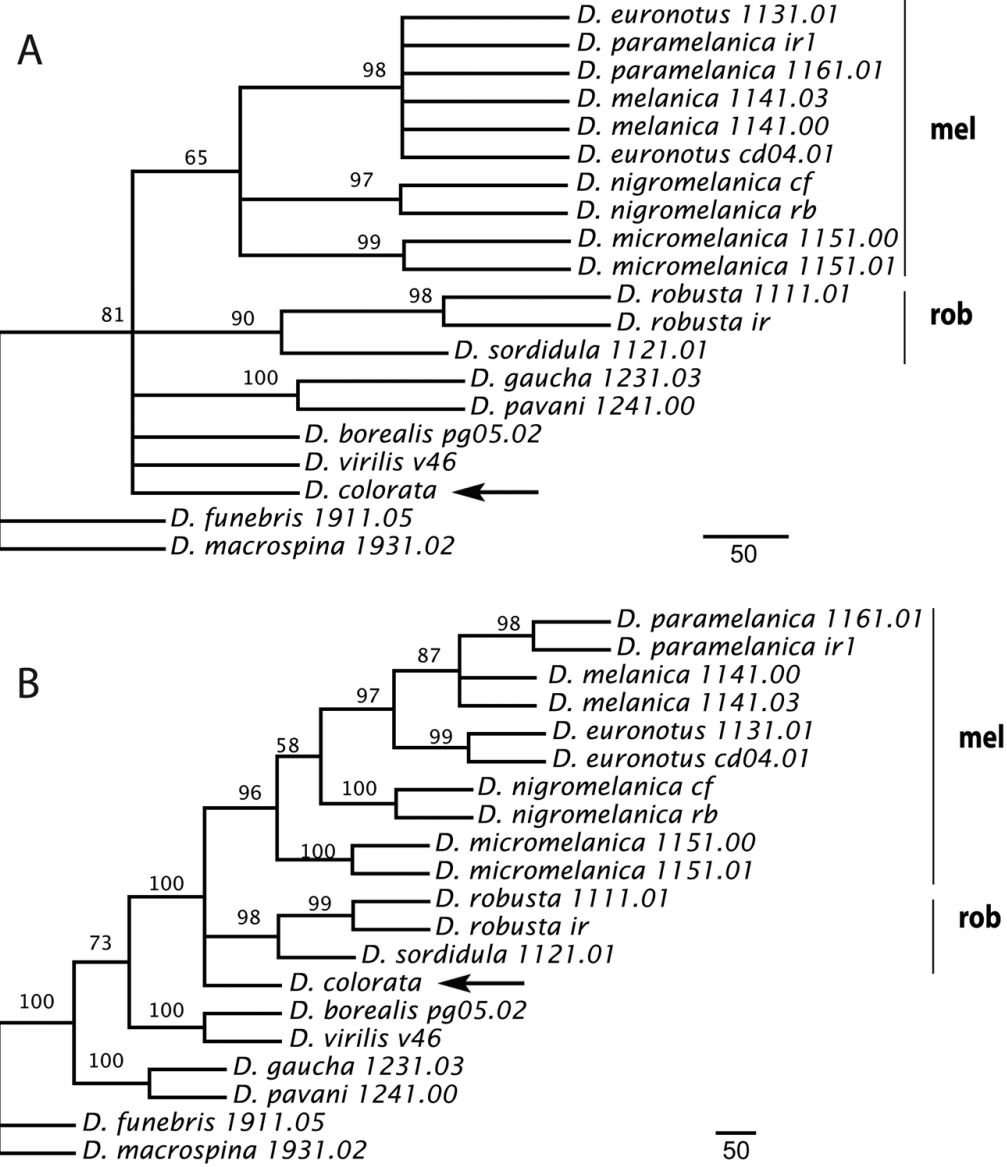
**Inferred trees derived from mitochondrial and nuclear genes**. Majority rule consensus trees obtained by maximum parsimony using a) mitochondrial (combined *CoI *and *CoII*) and b) nuclear (combined *cac *and *sc*) data partitions. Numbers at the nodes indicate bootstrap values from maximum parsimony (10^4 ^replicates). Scale bars indicate a branch length corresponding to 50 substitutions. Clades representing the *robusta *(rob) and *melanica *(mel) species groups are labeled.

**Figure 5:**
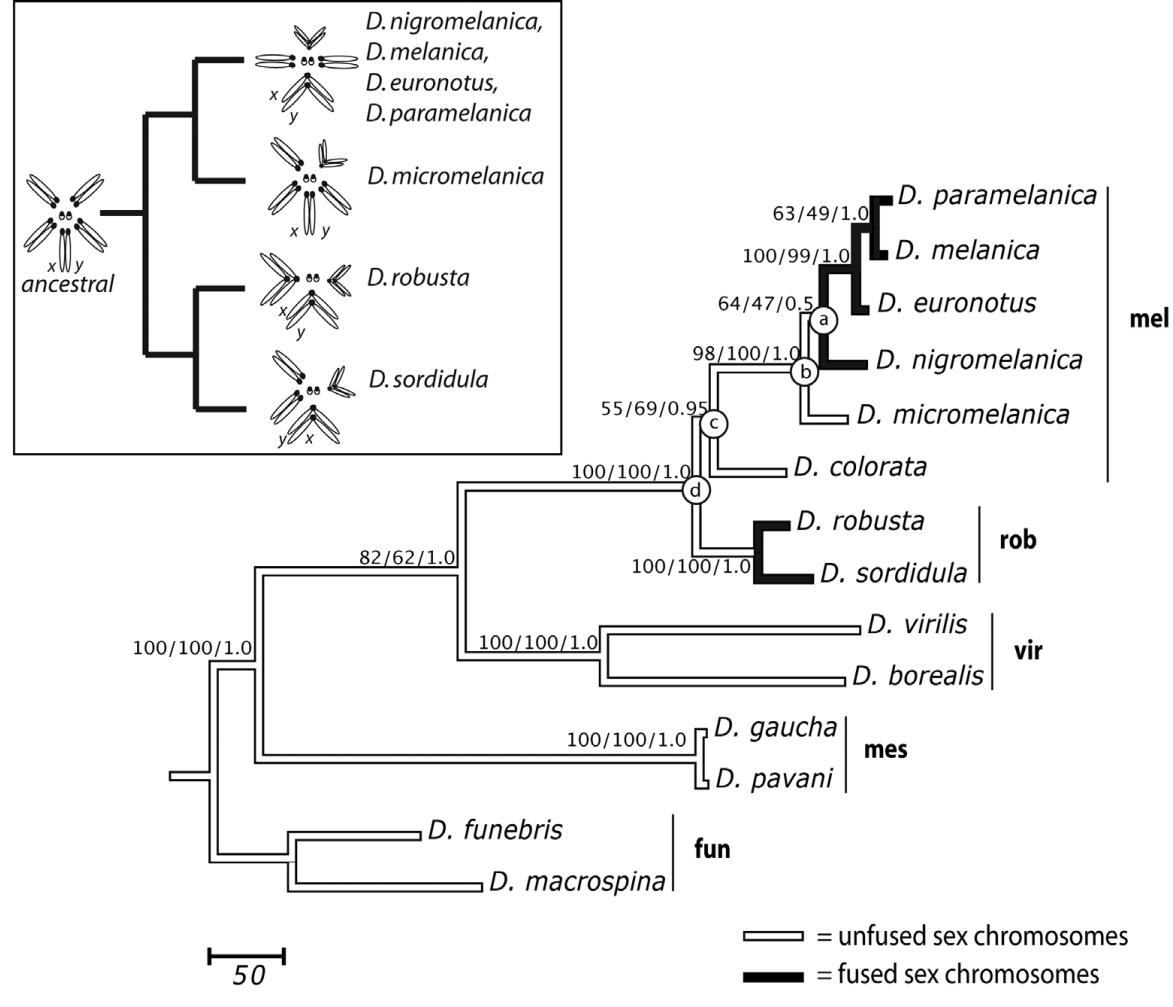
**Inferred tree using concatenated mitochondrial and nuclear genes**. Most parsimonious tree obtained from heuristic search using the concatenated *CoI*, *CoII*, *cac *and *sc *datasets. The numbers at the nodes indicate bootstrap values from maximum parsimony (10^4 ^replicates), maximum likelihood (10^3 ^replicates), and Bayesian posterior probability percentages (2 × 10^6 ^generations) respectively. Supports for within-species clades were all high and not included. Species groups are indicated: *melanica *(mel), *robusta *(rob), *virilis *(vir), *mesophragmatica *(mes) and *funebris *(fun). Inset shows the inferred history of the centric fusion in the *melanica *and *robusta *species group based on this phylogeny.

Maximum likelihood, maximum parsimony, and Bayesian methods all recovered similar tree topologies using the mitochondrial (Fig. 4A), nuclear (Fig. 4B) and combined (Fig. 5) data partitions. Trees from the concatenated mitochondrial genes contained several unresolved nodes at the level of relationships among species within groups (Fig. 4A). However, the nodes supporting the *melanica *and the *robusta *species groups, excluding *D. colorata*, were resolved with high confidence. Despite being currently recognized as a member of the *melanica *species group, *D. colorata *was placed basal to the *melanica *and *robusta *groups. The relationships among outgroups was poorly defined, even for the pair of species representing the two major phylads within the *virilis *species group.

The phylogenies derived from the concatenated nuclear genes exhibited high support at almost all nodes (Fig. 4B). The *robusta *and the *melanica *species groups were discriminated with high bootstrap support. *D. colorata *was again placed basal to the *robusta *and *melanica *groups but, as in the phylogeny obtained from mitochondrial genes, its position was not strongly supported. Importantly, *D. micromelanica*, a species with unfused sex chromosomes, was placed as the basal lineage within the *melanica *species group.

The trees from the analysis using the total concatenated data set did not display conflicts with those obtained from the separate mitochondrial and nuclear partitions (Fig. 5). Here, the *melanica *species group was monophyletic and well supported, and *D. colorata *was placed as the basal lineage, but with low bootstrap support. The *D. pavani *– *D. gaucha *cluster was placed basal to the *D. virilis*-*D. borealis *node, thus representing the *virilis *section of the *virilis*-*repleta *radiation, as shown previously [57].

Overall, our results indicate that the *melanica*-*robusta *clade contains two separate lineages with metacentric sex chromosomes arising from independent fusions with an autosomal element. One fusion is present in the karyotypes of four species belonging to the *melanica *species group (node *a *in Fig. 5) and the other is present in the two species contained within the *robusta *species group (node *d*). Two nodes (nodes *b *and *c*) that apparently represent the ancestral arrangement of the sex chromosomes separate these two lineages.

### Hypothesis test of the sex chromosome rearrangement

Contrasts between different topologies relevant to the origin of rearranged sex chromosomes in the *melanica *and *robusta *groups reject the hypothesis of a single origin. Three alternative topologies consistent with independent origins of rearranged sex chromosomes are each shown to be credible by the Shimodaira-Hasegawa (SH) and the approximately unbiased (AU) tests. Each of these topologies reflects alternative placements of *D. colorata *relative to the *melanica *and *robusta *groups. Forced monophyly of species having rearranged sex chromosomes corresponds with a significant reduction in likelihood score (Table 4). These differences in likelihood scores are also reflected in the tree lengths obtained from weighted parsimony. Overall, this comparison indicates that topologies disrupting monophyly of the *melanica *group, including *D. micromelanica *which maintains the ancestral arrangement of the sex chromosomes, are significantly incompatible with the data. Monophyly of all species containing a similar rearrangement of their sex chromosomes is, therefore, rejected in favor of topologies supporting the hypothesis of two independent rearrangements.

**Table 4:** Contrast Among Topologies Representing Alternative Hypotheses on the Origin of Rearranged Sex Chromosomes in the *melanica-robusta *clade

Topology	-lnL	Length	SH	AU
**Two Origins**				
(out, ((rob, sor), (col, (mic, (nig, (eur, (mel, par)))))))	7497.2	1231.4	0.884	0.725
(out, (col, ((rob, sor), (mic, (nig, (eur, (mel, par)))))))	7498.1	1242.4	0.694	0.410
(out, ((col, (rob, sor)), (mic, (nig, (eur, (mel, par))))))	7498.4	1243.6	0.649	0.170
**One Origin**				
(out, (col, (mic, ((rob, sor), (nig, (eur, (mel, par)))))))	7535.8	1286.5	0.002*	1E-4*
(out, (mic, (col, ((rob, sor), (nig, (eur, (mel, par)))))))	7540.6	1288.1	0.002*	2E-4*

Note: SH = P-value Shimodaira-Hasegawa test; AU = P-value approximately unbiased test; out = outgoup species; rob = *D. robusta*; sor = *D. sordidula*; col = *D. colorata*; mic = *D. micromelanica*; nig = *D. nigromelanica*; eur = *D. euronotus*; mel = *D. melanica*; par = *D. paramelanica*. Constrained topology of outgroup species relative to ingroup: ((*funebris*, *macrospina*), ((*pavani*, *gaucha*), ((*virilis*, *borealis*), (ingroup)))). Asterisk indicates topologies rejected at the 1% level.

### Estimated ages of the derived sex chromosomes

By estimating dates of divergence at specific nodes within the consensus tree, the intervals in which these two rearranged sex chromosomes arose were inferred. Both the rate of synonymous substitutions (Ks) and the penalized likelihood (PL) methods yielded similar estimates of the dates for the relevant nodes used to determine these intervals (Table 5). On the other hand, Bayesian dating (BD) yielded higher estimates of the dates for the four relevant nodes. In addition, confidence intervals were considerably broader with BD than with other methods.

**Table 5:** Estimated Ages of Sex-Chromosome Rearrangements in the *melanica *and *robusta *Species Groups

		Dating estimation methods * Calibration points			
Event		Ks mtDNA *Caletka and McAllister [60]	Ks nuclear *Bonacum et al [88]	PL * Throckmorton [46]	BD *Throckmorton [46]
**Fusion in the *melanica *group**	Minimum ^a^	7.0 (5.2 – 8.9)	8.1 (5.3 – 11.1)	7.5 (5.6 – 10.7)	8.4 (2.9 – 16.5)
	Maximum ^b^	8.2 (6.1 – 10.6)	9.5 (6.3 – 13.0)	8.9 (6.5 – 12.6)	13.3 (5.4 – 20.1)
**Fusion in the *robusta *group**	Minimum ^c^	8.0 (6.1 – 9.9)	9.9 (6.7 – 13.3)	10.1 (7.0 – 13.5)	14.7 (10.9 – 19.0)
	Maximum ^d^	11.1 (8.6 – 14.1)	15.1 (11.0 – 19.5)	15.6 (11.9 – 20.0)	20.6 (17.0 – 24.6)

Note: Values in parentheses indicate the 95% confidence intervals. Superscripts^a, b, c, & d ^indicate respective nodes in Figure 5. Dating methods: Ks, rate of synonymous substitutions; PL, penalized likelihood; BD, Bayesian dating. See text for details on calibration points.

Rapid divergence at the base of the *melanica *group constrains the timeframe in which the centric fusion in the group could have arisen. Origin of the centric fusion in the *melanica *group was estimated to have occurred between 7.5 and 9 Mya using the dates obtained by PL (Table 5). The maximum value of this interval is based on the estimated date of the divergence of *D. micromelanica*. The minimum value corresponds to the first instance of divergence within the clade sharing the apomorphic (fused) arrangement of the sex chromosomes, which corresponds to the divergence of *D. nigromelanica*.

On the other hand, low diversity in the *robusta *group provides little constraint on the age of this centric fusion, and a window of 10 to 15 Mya is estimated from the lineages represented in the analysis (Table 5). Uncertainty in the time of origin is due to the absence of a closely related basal lineage with unfused sex chromosomes and having only two species with fused sex chromosomes. Inclusion of a greater diversity of Asian representatives of the *robusta *group that also contain rearranged sex chromosomes [36,49] would undoubtedly increase the minimum age of this fusion, but should have little effect on the maximum.

## Discussion

Resolution of the phylogenetic relationships among species sharing similar rearrangements of sex chromosomes is critical for proposing and testing hypotheses about parallelism and convergence of evolutionary pathways underlying the differentiation of new sex chromosomes. This phylogenetic analysis of the *robusta *and *melanica *species groups, two closely related lineages, revealed two independent origins of a fusion between the sex chromosomes and an autosomal pair. Our results are consistent with centromeric fusion between an autosome and the X (and possibly the Y) occurring independently in both of these groups. The independent origin of each fusion is strongly supported by the consensus tree topology, inferred via different data partitions and methods, since the two clades containing rearranged sex chromosomes are separated by at least two nodes representing common ancestors with pleisiomorphic (unfused) sex chromosomes (Fig. 5). In agreement with the history of the chromosomal rearrangements depicted by the consensus topology, the alternative hypothesis of a monophyletic origin is rejected based on statistical criteria upon application of tests for contrasting phylogenetic hypotheses.

Given the topology of Figure 5, a monophyletic origin of the fused arrangement of the sex chromosomes and autosomes in the *robusta *and the *melanica *groups would be feasible only if reversal of the rearrangement through fission is considered plausible. Under this hypothesis, independent fissions of the derived sex chromosome arrangement are required in *D. colorata *and *D. micromelanica*. Overall, this hypothesis does not alter the topology, but it does require one additional instance of chromosomal rearrangement, entailing one fusion followed by two independent fissions, thus it is less parsimonious than the inference of two independent fusions within both the *melanica *and *robusta *species groups. Furthermore, taking into account that autosomal arms diverge through gene loss and dosage compensation after fusion with the sex chromosomes [9,26,58], reversion of this type of chromosomal rearrangement is generally considered unlikely. Consequently, the character states studied here (fused and unfused sex chromosomes) are treated as polarized. A more direct test of the plausibility of chromosomal fission in these groups would require a phylogenetic analysis including additional taxa contained in the *melanica-robusta *clade and having unfused sex chromosomes–coupled with better resolution of the relationships among *D. micromelanica*, *D. nigromelanica*, and the other species in the *melanica *group. By inference from other studies, the ancestral arrangement of the X in *D. longiserata *and *D. tsigana *[59], coupled with the placement of this species pair as a single clade arising from a basal node of the *melanica *group [49], further suggests fission as an unlikely mechanism for generating the observed karyotypic variability and implies the independent origin of the rearranged sex chromosomes in the *robusta *and *melanica *species groups. Preliminary phylogenetic analyses utilizing available sequences of *D. moriwakii *indicate it is a sister of *D. colorata*, so unfortunately, its placement does not provide an additional node to inform the history of the chromosomal forms, but it could help resolve the position of these species within the *melanica*-*robusta *clade (see below).

These analyses strongly supported monophyly of the traditionally recognized *melanica *species group (i.e., exclusive of *D. colorata*); however, topological relationships within the group are not strongly supported by any method. At the base of the *melanica *clade, the branching order for *D. micromelanica*, which has unfused sex chromosomes, and the branches containing species with fused sex chromosomes represented by *D. nigromelanica *and the lineage leading to the other species in the *melanica *group are poorly supported. Low support for these nodes, derived from nonparametric and Bayesian MCMC methods, may be due to insufficient phylogenetic signal indicated by the short internode distance (node *a *to *b*, Fig. 5). In a direct contrast, the constrained topology with *D. micromelanica *as the basal lineage for the *melanica *group was less than one step shorter (using a differential weighting scheme) than the topology constrained with *D. nigromelanica *as the basal lineage, thus these alternative topologies do not differ statistically (data not shown). Although the consensus topology obtained here is consistent with a single fusion of the sex chromosomes within the *melanica *species group, thus agreeing with the phylogeny of the *melanica *group initially proposed by Stalker [44], this topology was weakly supported by the analysis.

Placement of *D. nigromelanica *as the basal lineage for the *melanica *group is indicated by comparison of polytene chromosomes even though it conflicts with overall chromosome morphology [45]. In order to explain the existence of both fused and unfused sex chromosomes in derived nodes, Stalker proposed the maintenance of the sex-chromosome fusion as a polymorphism in the population from which the basal lineages of the *melanica *group arose. A similar type of polymorphism is currently observed in *D. americana *[30,60]. Under this hypothesis the fusion between sex chromosomes and autosomes predated the diversification of the *melanica *group, and fixation/loss occurred separately in different lineages. Although the resolved topology for the *melanica *group is inconsistent with this hypothesis, it cannot be rejected due to the tight clustering, and ultimately, weak support for each of the basal nodes. On the other hand, accounting for all cases of fused and unfused sex chromosomes for the entire *melanica*-*robusta *clade through ancestral polymorphism requires the maintenance of both chromosomal forms over a period of about 8 My (Node *d *to *a*, Fig. 5). Although a short-term polymorphism may account for conflicting phylogenetic signals among lineages in the *melanica *group, the later scenario seems unlikely since the hypothesis entails the maintenance of the polymorphism for an extended period of time represented by the split between the two groups until the initial diversification of the extant lineages within the *melanica *group.

Our analysis indicates uncertainty in the exact position of *D. colorata*. The contrasts among alternative topologies testing the number of fusion events within the *melanica-robusta *clade revealed that *D. colorata *could be placed either basal to the *melanica *clade, basal to the *robusta *clade, or basal to both (Table 4). Chromosomal morphology, including an unfused X chromosome, indicates that *D. colorata *is a sister species of D. *moriwakii *[44], and a recent molecular phylogeny placed *D. moriwakii *basal to the *robusta *group [49]. Taken together these observations indicate uncertainty in the phylogenetic position of a clade containing *D. colorata *and *D. moriwakii *and suggest the possibility that these species are contained in a separate clade originating near the split between the *melanica *and *robusta *species groups. Resolution of the phylogenetic position of *D. colorata *and *D. moriwakii *as originating from a basal node in either the *melanica *or *robusta *groups would further clarify the evolutionary history of these alternatively arranged sex chromosomes.

Interpretations of the ages of these chromosomal rearrangements are presented in the context of the Ks and PL estimates, since the BD method yielded larger values with considerably broader confidence intervals. The current taxa broadly constrain the estimated age of the centromeric fusion in the *robusta *group. This rearrangement could have arisen in an interval from 10 to 15 mya constrained by the time of divergence between *D. robusta *and *D. sordidula *to the split between the *robusta *and *melanica *groups (Fig 5, Table 5). Origin of the fusion in an Asian ancestor prior to the divergence and dispersal of *D. robusta *into North America is indicated [49], but this suggests a more recent date than the 20–25 My previously inferred for this dispersal event [61]. Conversely, origin of the fusion in the *melanica *group is tightly constrained within the consensus topology. The estimated age of fusion in the *melanica *group is constrained to a minimum of 7.5 My, corresponding to the divergence of *D. nigromelanica*, and a maximum of 9 My, corresponding to the initial divergence within the group. This chromosomal rearrangement apparently occurred following the dispersal of the ancestor of this North American clade from Asia [49]. Diversification of the subgroup comprising *D. melanica*, *D. euronotus *and *D. paramelanica *was dated at 2.2 mya (CI 1.33 – 3.91, PL analysis). Therefore, newly derived sex chromosomes in the *melanica *group are currently represented by two distinct lineages (*D. nigromelanica *and the "*melanica *subgroup"), which diversified near the point when this rearrangement arose in their common ancestor. Considering this history and extant biodiversity, the new chromosomal arms fused to sex chromosomes in the *melanica *group represent an excellent substrate for exploring patterns of parallel evolution arising from divergence of the newly sex-linked regions.

Although our results do not confirm the identities of the chromosomal arms fused with the sex chromosomes, independent fusions putatively involving the same chromosomal element in both species groups represents a case of extreme convergence. Additional cases of translocations to the sex chromosomes are reported for species contained within the *virilis*-*repleta *radiation, which represents one of the major clusters of species diversity within the genus *Drosophila*. Other than the rearrangement of the X chromosome of *D. americana *[60], these newly derived regions of the sex chromosomes have not been examined very extensively. Phylogenetic relationships of the *virilis *section obtained by Wang et al [49] and karyotypes described by He *et al*. [62] indicate that the karyotype of *D. lacertosa *represents at least one additional case of fusion between an autosome and the sex chromosomes in a lineage closely allied with the *melanica*-*robusta *clade. Additionally, two members of the *repleta *species group, *D. canalinea *and *D. castanea*, have been reported as having sex chromosomes fused with autosomal elements [37,38]. In the phylogeny of Robe *et al*. [57], *D. canalinea *represents a basal lineage of the *repleta *section within the *virilis*-*repleta *radiation and the phylogenetic position of *D. castanea *is currently unknown. Overall, the fusions with the sex chromosomes represented by *D. americana *(B element), *D. lacertosa *(D element), *D. canalinea *(D element), *D. castanea *(B element) and the two analyzed in this study of the *melanica *and *robusta *species groups (both D element), delineate six independent fusion events involving either the B or D elements in the *virilis-repleta *radiation, a clade that is estimated to have originated around 36 mya during the late Eocene [46].

Currently, sex chromosome evolution in the genus *Drosophila *has been investigated in a set of species representing disparate time points since the origin of the rearrangements that form the newly sex-linked regions. The youngest rearrangement is the fusion between the X chromosome and an autosome in *D. americana*, which appears to have originated less than 0.5 mya [60] and is still segregating in populations with the ancestral arrangement [30]. A slightly older (~1 My), but clearly more diverged, newly sex-linked region is present in *D. miranda *[63]. A fusion involving the sex chromosomes within *D. albomicans *may represent an intermediate between these two points [64]; however, the extent of divergence between the newly X-linked and Y-linked regions is currently unknown. The derived component of the sex chromosomes in *D. pseudoobscura *is completely heteromorphic and the causative rearrangement has been estimated within a broad window of 6 to 10 mya [65]. This analysis suggests an older age for the origin of the newly sex-linked arm in *D. robusta*, so given the recruitment of the dosage compensation machinery along the newly derived arm of the X in both of these species [22,23], a period of less than 10 My appears sufficient for a complete transition to a heteromorphic state in the absence of male crossing over. Narrow constraints on the origin of the rearranged sex chromosome in the *melanica *group, followed quickly by the divergence of two lineages containing this rearrangement, makes this newly sex-linked region especially appealing for further analyses of temporal patterns of divergence. Synapomorphic changes within the newly sex-linked region of *D. nigromelanica *and, for example, *D. euronotus *would reveal early events in the transition from autosome to sex heteromorphic sex chromosomes, whereas autapomorphic changes in these species would reveal later events in this transition. However, these studies will ultimately require direct assessment of regions isolated from the neo-sex chromosomes.

## Conclusion

The *robusta *and *melanica *species groups are two closely related clades in the genus *Drosophila *containing independent rearrangements of their sex chromosomes. Ages of the chromosomal fusion events responsible for generating the newly sex-linked arms in both groups are quite old, consistent with previous studies indicating acquisition of dosage compensation on the new arm of the X within *D. robusta*. Diversification in the melanica group near the time of origin of its newly sex-linked region generated independent lineages in which the transition from autosomal pair to heteromorphic sex chromosome may have proceeded in parallel. The phylogenetic context established by this analysis provides a framework for comparative studies of sex chromosome evolution.

## Methods

### Flies

Flies were either obtained from recently collected material maintained as iso-female lines or from the Tucson *Drosophila *Stock Center (Tucson, AZ). When possible, two different strains of each ingroup taxon were included in the analysis. The source of each line used in the analysis is listed in Table s1 (see Additional file 1). Species identification of collected material was determined by morphological examination using a taxonomic key to the United States species of *Drosophila *[66]. Species represented in the analysis and previously unavailable in a public repository have been deposited in the Tucson *Drosophila *Stock Center.

A broad sample of outgroup taxa, representing the ancestral arrangement of the sex chromosomes, was also included in the analysis. Sequences of *D. funebris *and *D. macrospina*, representing the *funebris *species group, which is basal to the *virilis*-*repleta *radiation [47,57], were used as a distant outgroup to define the root in the phylogenetic trees. Also, *D. pavani *and *D. gaucha*, belonging to the *mesophragmatica *species group, were used as representatives of an early bifurcation of the *virilis*-*repleta *radiation [57]. *D. virilis *and *D. borealis *were included as representatives of the *virilis *species group, which is closely related to the *robusta*-*melanica *clade [47,49,57]. This sampling strategy allowed the use of alternative biogeographic assumptions in the dating estimation of the origin of the centric fusions (see below).

### Karyotype analysis

Metaphase chromosomes were obtained from third-instar larval ganglia following the method of Pimpinelli [67]. Briefly, ganglia were dissected in 0.7% sodium chloride and then transferred to a hypotonic treatment by incubating in 0.5% sodium citrate for 10 min. The ganglia were fixed for 10 to 20 seconds in 3:1 ethanol:acetic-acid and transferred to 4 μl of 45% acetic acid on a siliconized coverslip. Finally, each ganglion was squashed and preps were frozen in liquid nitrogen for about 1 minute. Coverslips were removed and the preparations were dehydrated in 95% ethanol for 10 minutes. Slides were stained with 4'-6-Diamidino-2-phenylindole (DAPI) and mounted in glycerol. Metaphase chromosomes from males and females were digitally imaged and compared to reveal the morphology of the sex chromosomes.

Sex chromosomes were identified by comparing the karyotypes of males and females to detect heteromorphism between X and Y chromosomes. In particular, differential degree of DAPI-staining was used as a morphological marker of sex chromosomes, since Y chromosomes typically exhibit high affinity for DAPI due to enrichment in AT-rich heterochromatin [68,69].

### DNA extraction, amplification and sequencing

A single fly from each line was homogenized and DNA was extracted using the DNeasy Tissue Kit (Qiagen). PCR amplification was performed by mixing 1.0 μl of extracted DNA with 49 μl of PCR master mix. Reaction conditions for the PCR were 1× reaction buffer, 0.1 mM each dNTP, 0.2 μM each primer, and 2.5 units of Taq Polymerase (New England Biolabs). Table s2 (see Additional file 2) summarizes the primer sequences, annealing temperatures and fragment sizes of the PCR products used to obtain the sequences of mitochondrial genes *Cytochrome oxidase I *(*mtCoI*) and *Cytochrome oxidase II *(*mtCoII*), and for nuclear X-chromosome gene regions of *cacophony *(*cac*) and *scute *(*sc*). An MJ Research thermocycler was used to incubate the reactions for 2 min at 95°C, and cycle 35 times at 95°C for 0.5 min, 0.5 min at the annealing temperature and 72°C for 1 min followed by 10 min at 72°C. PCR product was purified with the MinElute PCR Purification Kit (Qiagen).

Purified PCR products were sequenced with Big Dye Terminator Chemistry V3 (ABI). 5–10 ng of amplified target was added to 4.5 μl of Big Dye reaction mix with 0.4 μM of primer to a total volume of 10 μl. Reactions were ramped to 96°C at 2.5°C/sec and cycled 30 times for 10 sec at 96°C, 5 sec at 50°C and 2 min at 60°C. Sequences were cleaned using Wizard^(*r*) ^Magnesil Green Sequencing Reaction Clean-Up System (Promega) and analyzed with an ABI 3730. Sequences were trimmed and edited using Sequencher 3.1 (Gene Codes).

### Phylogenetic analysis

Sequences of the mitochondrial genes were aligned using ClustalX 1.81 [70] using default parameters. The nuclear genes contained many indels within coding regions, so following the removal of introns, the exon regions were aligned against the respective alignment of amino acid sequences using ClustalW as implemented in DAMBE 4.2.13 [71]. Nucleotide alignments were corrected by hand using BioEdit [72]. Sites lacking clear evidence of homology due to indels of codon repeats, as observed in *sc*, were excluded from the analyses. Saturation at third codon positions was inspected by plotting the uncorrected distances of transitions and transversions against GTR + Γ pairwise distances estimated using PAUP 4.0b10 [73]. Codon positions with significant saturation were removed to reduce noise in the phylogenetic signal.

In order to test for congruence among data partitions, 10^4 ^replicates of the partition homogeneity test (ILD) [74,75] were run in PAUP 4.0b10, comparing the phylogenetic signal within each of the following partitions: a) between both mitochondrial genes, b) between both nuclear genes, c) between mitochondrial versus nuclear genes, and d) among all genes.

Phylogenetic analyses were executed in PAUP 4.0b10 for maximum parsimony (MP) and maximum likelihood (ML), and in MrBayes 3.0b4 [76] for Bayesian analysis (BA). Optimal models of nucleotide substitution supported by the Akaike information criterion test (AIC) [77] as implemented in ModelTest [78,79] were used in ML and BA. Parameters for the priors of topology inference, used in BA, were tested and selected using MrModeltest 2.0 [80]. The confidence values for each clade in MP and ML were assessed by bootstrap [81] with 10^3 ^pseudoreplicates, heuristic searching, and random-addition of sequences. MP was assessed with differential weights for transitions and transversions, using the values of those parameters as estimated by ModelTest. In the BA analysis, the run was conducted for 10^6 ^generations in four independent chains. The generations needed to reach the stationary state were evaluated by plotting the likelihood values (-lnL) for 10^4 ^sampled trees. Only generations from the stationary period were included in the computation of the consensus tree, applying the 50% majority rule.

### Hypothesis testing

A contrast among five plausible topologies bearing on the origin of the rearranged sex chromosomes was performed. The contrast included three constrained topologies consistent with independent origins of rearranged sex chromosomes in the *melanica *and *robusta *species groups, and two topologies consistent with a single monophyletic origin of rearranged sex chromosomes. Although the general contrast relating to alternative hypotheses regarding the origin of the rearranged sex chromosomes was specified *a priori*, the specific topologies used from this analysis were based upon and included the maximum likelihood tree, and therefore, the comparisons represent a mix of *a priori *and *a posteriori *hypotheses (see Goldman et al [82] for statistical issues arising from such contrasts).

Contrasts were obtained using the approximately unbiased (AU) [83] and Shimodaira-Hasegawa (SH) [84] tests performed in CONSEL ver. 0.1i [85]. Site-wise log-likelihoods were estimated for the five alternative topologies using PAUP* under the GTR+I+Γ substitution model. The credible set of topologies was determined using the default parameters in the program makermt of the CONSEL package to obtain *P*-values derived from 10 sets of 10,000 bootstrap replicates of the likelihood matrix.

### Dating estimation

The molecular clock was tested using the parameters estimated in Modeltest (see Results). Likelihood ratio tests [86] did not reject the occurrence of a molecular clock when mitochondrial or nuclear loci from the complete data set were analyzed (CoI – CoII: -2ln Δ = 12.54, 18 df, P = 0.82; *cac – sc*: -2ln Δ = 28.56, 18 df, P = 0.054). However, the molecular clock was rejected when only the *melanica *and *robusta *groups (the focal groups of this study) were considered (CoI – CoII: -2ln Δ = 86.32, 18 df, P < 0.01; *cac – sc*: -2ln Δ = 838.47, 18 df, P < 0.01). Therefore, the age of the centric fusions was estimated assuming a molecular clock and with two relaxed methods not dependent on this assumption.

Clock-based dating estimations were performed using the concatenated sequences of the nuclear genes and the Kimura 2-parameter distances for synonymous substitutions (Ks) as implemented in K-Estimator [87]. For the molecular clock calibration we used a rate of synonymous substitution of 7.95 × 10^-9 ^substitutions per year, which was obtained from analysis of Adh sequences including 17 species belonging to the planitibia group with *D. picticornis *as the outgoup (Genbank Accessions AY006408, AY006410 – AY006425). Origin of the *planitibia *group was dated at 6.1 mya by Bonacum et al [88] using levels of molecular divergence and multiple geological calibration points.

The divergence among mitochondrial sequences was compared in a second analysis of dating estimation based on Ks distances. Caletka and McAllister [60] proposed 3 Mya for the divergence between *D. lummei *and the common ancestor of the North American members of the *virilis *group based on biogeographic and paleoclimatic evidence. Therefore, we estimated the Ks divergence between *D. lummei *and the *D. americana*-*D. novamexicana *clade using *mtCoI *in order to obtain an estimate of the substitution rate of the mtDNA.

Despite the potential violation of the molecular clock, divergence of synonymous substitutions was used for dating estimation because it permitted us to use a different set of assumptions (rates of divergence, biogeographic milestones and models of nucleotide substitutions) than two relaxed molecular clock methods: penalized likelihood and Bayesian dating estimation.

Penalized likelihood (PL) allows variation in rates along branches was assessed using r8s version 1.7 [89]. First, cross validation was performed to estimate the optimum value of the smoothing parameter λ, by using the truncated-Newton method. The gamma shape estimated via Modeltest for the concatenated data set was used as input for this analysis. The *virilis*-*borealis *clade was pruned prior to rate and divergence time estimations.

Estimation of confidence for node ages was assessed by two different approaches. First, 10^3 ^trees were generated by bootstrapping the data matrix and obtaining branch lengths for the constrained consensus topology using maximum likelihood. The resulting trees were used as separate inputs for r8s. Second, post-burnin trees, sampled each 250 generations, were obtained using MrBayes and filtered with PAUP to obtain a sample of 1,105 trees differing in branch length but containing the consensus topology. The first approach has the inconvenience that only one model of nucleotide substitution is applied to the concatenated matrix. As pointed out by Schwarz et al [90], the second strategy overcomes that difficulty, but is exposed to the effect of dependence of parameters among trees, since the MCMC algorithms modify only a few parameters per generation. We set the prior for the mean of the origin of the *virilis*-*repleta *radiation at 36 Mya, as suggested by Throckmorton [46] based on the present distribution following the disjunction of tropical and temperate floras in North America, which occurred during the late Eocene, and supported by the survey of molecular divergence in *Drosophila *by Beverley and Wilson [91].

Bayesian dating (BD) was performed as implemented in Multidivtime, which allows multiple data partitions [92,93]. By using a MCMC algorithm, the posterior distributions of rates and divergences were obtained by specifying different substitution models for each partition. Four partitions were used in this analysis: *CoI *(1st and 2nd codon positions), *CoII *(1st and 2nd codon positions), *cac *and *sc*. Parameters for the F84 + γ model, the most parameter-rich model implemented in Multidivtime, were estimated using PAML 3.14 [94]. Maximum likelihood and variance-covariance matrix of the branch lengths were estimated using the Estbranches program. Multidivtime was used to obtain the posterior distribution of divergence dates and substitution rates for each node. MCMC was performed by running 10^6 ^generations, with sampling frequency every 100 generations and a burn-in period of 10^5^. Confidence in node ages was assessed using 95% credibility intervals. The posterior distribution analyses were run ten times and the congruence among results was checked in order to ensure the Markov chain reached stationarity. The calibration point was the same as used in the PL method.

## Authors' contributions

SVF carried out the cytogenetic and phylogenetic studies and wrote the manuscript. ALE carried out the sequencing and assisted in writing and editing the manuscript. BFM conceived and coordinated the study, contributed to the design, collected specimens, and contributed to the writing and editing the manuscript. All authors read and approved the final manuscript.

## Supplementary Material

Additional File 1 Descriptions of *Drosophila *strains with GenBank accessions of each sequence. The table lists the source and locality information for each strain used in the analysis, and includes the GenBank accession number of each sequence. Click here for file

Additional File 2 Primers, Annealing Temperatures (T_a_) and PCR Product Size of Gene Regions. The table lists the primer sequences, annealing temperature and product size for each gene region examined in the analysis. Click here for file
