# Supplementary material for: Independent Origins of New Sex-Linked Chromosomes in the melanica and robusta Species Groups of Drosophila
Source: BMC Evol Biol. 2008 Jan 29;8:33. doi: 10.1186/1471-2148-8-33 (PMC2268673; doi:10.1186/1471-2148-8-33)
Supplement: Additional File 1 — Descriptions of Drosophila strains with GenBank accessions of each sequence. The table lists the source and locality information for each strain used in the analysis, and includes the GenBank accession number of each sequence. [file 1471-2148-8-33-S1.doc]

**TABLE s1 - Descriptions of *Drosophila*** Strains with GenBank Accessions of each Sequence

| ***Strain*** | ***Species group*** | **Locality** | **Genbank Accession** | | | |
| --- | --- | --- | --- | --- | --- | --- |
|  |  |  | **CoI** | **CoII** | **cac** | **sc** |
| *D. euronotus* 1131.01^TSC^ | melanica | Tallahassee, FL (USA) | EU390738 | EU390742 | EU390702 | EU390762 |
| *D. euronotus* cd04-1 | melanica | Columbia, LA (USA) | EU390727 | EU390743 | EU390703 | EU390763 |
| *D. melanica* 1141.00 ^TSC^ | melanica | Myakka Head, FL (USA) | EU390721 | EU390748 | EU390708 | EU390768 |
| *D. melanica* 1141.03 ^TSC^ | melanica | Austin, TX (USA) | EU390737 | EU390749 | EU390709 | EU390769 |
| *D. micromelanica* 1151.00 ^TSC^ | melanica | Coronado Nat. Park, AZ (USA) | EU390723 | EU390750 | EU390710 | EU390770 |
| *D. micromelanica* 1151.01 ^TSC^ | melanica | Smithville, TX (USA) | EU390722 | EU390751 | EU390711 | EU390771 |
| *D. nigromelanica* cf | melanica | Rend Lake, IL (USA) | EU390735 | EU390752 | EU390712 | EU390772 |
| *D. nigromenalica* rb | melanica | Ross Barnett Res, MS (USA) | EU390739 | EU390753 | EU390713 | EU390773 |
| *D. paramelanica* ir1 | melanica | Iowa River, IA (USA) | EU390736 | EU390755 | EU390715 | EU390775 |
| *D. paramelanica* 1161.01 ^TSC^ | melanica | Lake Champlain, VT (USA) | EU390729 | EU390754 | EU390714 | EU390774 |
| *D. colorata* bb | melanica | Reelfoot Lake, TN (USA) | EU390724 | EU390741 | EU390701 | EU390761 |
| *D. robusta* 1111.01 ^TSC^ | robusta | Lake Champlain, VT (USA) | EU390726 | EU390757 | EU390717 | EU390777 |
| *D. robusta* ir | robusta | Iowa River, IA (USA) | EU390725 | EU390758 | EU390718 | EU390778 |
| *D. sordidula* 1121.00 ^TSC^ | robusta | Sapporo, Hokkaido (Japan) | EU390728 | EU390759 | EU390719 | EU390779 |
| *D. borealis* pg | virilis | Poygan Marsh, WI (USA) | EU390720 | EU390740 | EU390700 | EU390760 |
| *D. virilis* | virilis | - | - | - | - | - |
| *D. pavani* 1241.00 ^TSC^ | mesophragmatica | Campos do Jordan (Brazil) | EU390732 | EU390756 | EU390716 | EU390776 |
| *D. gaucha* 15070-1231.03 ^TSC^ | mesophragmatica | Mendoza (Argentina) | EU390733 | EU390745 | EU390705 | EU390765 |
| *D. funebris* 1911.05 ^TSC^ | funebris | Espinoza, NM (USA) | EU390731 | EU390744 | EU390704 | EU390764 |
| *D. macrospina* 1931.02 ^TSC^ | funebris | Sonora (Mexico) | EU390730 | EU390747 | EU390707 | EU390767 |

Note: TSC indicates strains obtained from the Tucson *Drosophila* Stock Center. Nucleotide sequences of *D. virilis* were obtained from the whole genome sequence (Agencourt Bioscience Corporation) through FlyBase.
