# Supplementary material for: Independent Origins of New Sex-Linked Chromosomes in the melanica and robusta Species Groups of Drosophila
Source: BMC Evol Biol. 2008 Jan 29;8:33. doi: 10.1186/1471-2148-8-33 (PMC2268673; doi:10.1186/1471-2148-8-33)
Supplement: Additional File 2 — Primers, Annealing Temperatures (Ta) and PCR Product Size of Gene Regions. The table lists the primer sequences, annealing temperature and product size for each gene region examined in the analysis. [file 1471-2148-8-33-S2.doc]

TABLE s2 - Primers, Annealing Temperatures (Ta) and PCR Product Size of Gene Regions

| Locus | Primer ID | Sequence 5'-->3' | Ta (^o^C) | Product Size |
| --- | --- | --- | --- | --- |
| *COI* | LCO1490 | ggt caa caa atc ata aag ata ttg g | 52 | 658 bp |
|  | HCO2198 | taa act tca ggg tga cca aaa aat ca | 52 |  |
| *COII* | FOXI2 | aat atg gca gat tag tgc aa | 52 | 743 bp |
|  | ROXI2 | cca gta ctt gct ttc agt ca | 52 |  |
| *cac* | cac-F4 | cga aac ccc aag gca gtt g | 54 | 651-800 bp |
|  | cac-R4 | gcc ggt tag gat ttg aaa cac | 54 |  |
|  | cac-f5* | cga gtc gtc gtt taa ccg | 54 | - |
|  | cac-r3* | tcg aat atc gaa cca cta atg ac | 54 |  |
| *sc* | scute-F | cgc tat cag cac ata atg cc | 54 | 730-860 bp |
|  | scute-R | cga gtc gaa gga cat gc | 54 |  |
|  | scute-f2* | gtc aag cag gtg aac aa | 54 | - |
|  | scute-r3* | tgt tgc tgc tgc agc g | 54 |  |

Note: Primers for *CoI* are those proposed by Folmer *et al.* [1]. Asterisks indicate internal primers used for sequencing.

1. Folmer O, Black M, Hoeh W, Lutz R, Vrijenhoek R: **DNA primers for the amplification of mitochondrial cytochrome c oxidase subunit I from diverse metazoan invertebrates.** *Mol Mar Biol Biotechnol* 1994, **3**:294–299.
